# Seminal Plasma Modifies the Transcriptional Pattern of the Endometrium and Advances Embryo Development in Pigs

**DOI:** 10.3389/fvets.2019.00465

**Published:** 2019-12-18

**Authors:** Cristina A. Martinez, Josep M. Cambra, Inmaculada Parrilla, Jordi Roca, Graça Ferreira-Dias, Francisco J. Pallares, Xiomara Lucas, Juan M. Vazquez, Emilio A. Martinez, Maria A. Gil, Heriberto Rodriguez-Martinez, Cristina Cuello, Manuel Álvarez-Rodriguez

**Affiliations:** ^1^Faculty of Veterinary Medicine, International Excellence Campus for Higher Education and Research “Campus Mare Nostrum”, University of Murcia, Murcia, Spain; ^2^Institute for Biomedical Research of Murcia (IMIB-Arrixaca), Campus de Ciencias de la Salud, Murcia, Spain; ^3^Department of Clinical and Experimental Medicine (IKE), Linköping University, Linköping, Sweden; ^4^Department of Morphology and Function, University of Lisbon, Lisbon, Portugal

**Keywords:** seminal plasma, endometrium, transcriptome, embryo, preimplantation, pig

## Abstract

**Background:** Seminal plasma (SP) promotes sperm survival and fertilizing capacity, and potentially affects embryo development, presumably via specific signaling pathways to the internal female genital tract.

**Objectives:** This study evaluated how heterologous SP, infused immediately before postcervical artificial insemination (AI) affected embryo development and the transcriptional pattern of the pig endometria containing embryos.

**Materials and Methods:** Postweaning estrus sows (*n* = 34) received 40-mL intrauterine infusions of either heterologous pooled SP or Beltsville Thawing Solution (BTS; control) 30 min before AI of semen extended to 10% of homologous SP. Embryos (all sows) and endometrium samples (3 sows/group) were removed during laparotomy 6 days after the infusion of SP or BTS to morphologically evaluate the embryos to determine their developmental stage and to analyze the endometrial transcriptome using microarrays (PORGENE 1.0 ST GeneChip array, Affymetrix) followed by qPCR for further validation.

**Results:** Embryo viability was equal between the groups (~93%), but embryo development was significantly (*P* < 0.05) more advanced in the SP-treated group compared to control. A total of 1,604 endometrium transcripts were differentially expressed in the SP group compared to the control group. An enrichment analysis showed an overrepresentation of genes and pathways associated with the immune response, cytokine signaling, cell cycle, cell adhesion, and hormone response, among others.

**Conclusions:** SP infusions prior to AI positively impacted the preimplantation embryo development and altered the expression of the endometrial genes and pathways potentially involved in embryo development.

## Introduction

The relevance of keeping a proportion of seminal plasma (SP) in insemination doses to achieve acceptable fertility in the artificial insemination (AI) of pigs was a basic early conclusion of Russian pioneers (1). Since then, studies focused on the role of SP in transporting and protecting spermatozoa have included the design of chemically defined artificial SP [OLEP (2), Predil MR-A (3), etc.]. However, SP actions are wider, influencing the transient inflammation that semen deposition elicits in swine (4–6). Studies in rodents and pigs have suggested SP also impacts fertilization, implantation and pregnancy, modulating the local immunity of the female reproductive tract (7–9). Regulatory components in SP have the potential to alter the expression of active molecules in the female genital tract causing molecular, biochemical and cellular changes in the uterus through particular pathways (10). SP infusion has been recently shown to induce changes in the expression of immune-related genes in the reproductive tract of peri-ovulating pigs, indicating its very early signaling during mating/AI (11). However, we do not know if this signaling affects later events, particularly during the development of embryos preimplantation.

The most substantial evidence regarding the effects of SP on embryonic developmental competence comes from findings in rodents, in which SP infusion supports embryonic development and implantation (12, 13). In the absence of SP, fertilization and embryo development rates decrease, and fetal postimplantation losses increase (14, 15). Moreover, in mice and rats, embryo transfer (ET) protocols generally use recipients exposed to SP during estrus since it is fundamental for proper implantation and embryonic development (16). Despite such evidence, we still lack studies on endometrial molecular changes in response to SP infusions prior to AI and knowledge of how these alterations would influence the progression of porcine preimplantation embryos, a matter of utmost relevance not only for AI but also for other reproductive biotechnologies such as ET.

Therefore, the aims of this study were to examine the effects of heterologous SP infusions prior to AI on (i) changes in the global transcriptome of preimplantation endometrium and (ii) the development and viability of porcine preimplantation embryos.

## Materials and Methods

### Animals

All experimental procedures were assessed according to the 2010/63/EU EEC Directive for animal experiments and were revised and approved by the Ethical Committee for Experimentation with Animals of the University of Murcia, Spain (research code: 638/2012).

Crossbred sows (Landrace × Large-White), parity 2–7, with a lactation period of 21–24 days, were randomly selected at weaning and used for this experiment. The females were allocated into individual crates in a mechanically ventilated confinement facility at a commercial farm (Agropor SA, Murcia, Spain). The semen donors were sexually mature boars (2–3 years of age) of proven fertility, housed in climate-controlled individual pens (20–25°C) at a commercial breeding boar station for AI-dose semen production in Murcia (AIM Iberica, Spain). The animals had access to water *ad libitum* and were fed commercial diets according to their nutritional requirements.

### Experimental Design

To evaluate the effects of additional heterologous SP-infusions on the endometrial transcriptome and embryo development, a total of 34 postweaning estrus sows received 40-mL intrauterine infusions of either heterologous, pooled SP (*N* = 16) or Beltsville Thawing Solution [BTS; (17)] (control group; *N* = 18) 30 min before each AI. At day 6 of the cycle (Day 0 = onset of estrus), sows were subjected to laparotomies to immediately evaluate the effect of SP infusions on embryonic development stage and embryo quality. Additionally, uterine samples from 3 randomly selected embryo-bearing sows were collected for histological and transcriptomic analysis.

Samples were obtained from two different locations of a uterine horn, distal (ad-cervical, i.e., next to the uterine body) and proximal (ad-ovarian, i.e., next to the utero-tubal junction) and processed as described below. Endometrial histology was screened in each of the two localizations of each sow, examining a total of four slides per localization and sow. For transcriptomic analysis, a total of 12 microarrays were performed (six microarrays for SP-treated sows and six for control sows; three for each region, distal and proximal). To validate the microarray results, 15 transcripts were selected, and gene expression was analyzed by qPCR. For this validation, three biological replicates (three sows) per region of the endometrium and three technical replicates per sample were performed.

### Superovulation, Estrus Detection, and Insemination

The superovulation of donors was induced by the intramuscular administration of 1,000 IU equine chorionic gonadotropin (eCG; Folligon, Intervet, Boxmeer, The Netherlands) 24 h after weaning. Detection of estrus was performed by snout-to-snout contact of females with vasectomized mature boars while applying back-pressure to test for standing estrus reflex twice daily, beginning 1 day after weaning. Only sows with clear signs of estrus at 72–96 h posteCG administration were further intramuscularly administered 750 IU of human chorionic gonadotropin (Veterin Corion, Divasa, Farmavic S.A., Barcelona, Spain) at the first signs of estrus. Sows were postcervically inseminated 6 and 24 h after the onset of estrus with 1.5 × 10^9^ spermatozoa in 40-mL doses prepared with semen from adult boars extended with BTS to 10% (v/v) of homologous SP (18) (i.e., around 5 mL of semen extended with 35 mL of BTS). A total of three boars was used and within each replicate a similar number of sows (n=6) from the two groups was inseminated with sperm doses from the same boar.

### Ejaculate Collection and Seminal Plasma Preparation

Heterologous SP was obtained from full ejaculates without the gel fraction, which were collected from 16 healthy boars with known fertility using the gloved-hand method. These boars were different from those used for AI. Ejaculates were transported to the laboratory within 2 h after collection and centrifuged three times at 1,500 × g at 17°C for 10 min. The last SP-supernatant recovered was microscopically verified as sperm-free. SP from at least eight boars was then pooled, separated into aliquots of 40 mL and stored at −20°C until use. Immediately before their use, the SP doses were thawed at 37°C for 20 min and introduced into the uterine body of each sow by using a postcervical catheter.

### Embryo and Tissue Collection

Sows were sedated with azaperone i.m. (Stresnil®, Landegger Strasse, Austria; 2 mg/kg body weight, intramuscular) and anesthetized with sodium thiopental (B.Braun VetCare SA, Barcelona, Spain; 7 mg/kg body weight, intravenous) administered in the marginal vein of an ear. Isofluorane (IsoFlo®, Madrid, Spain; 3–5% in air) was used to maintain anesthesia, and buprenorphine (Buprex®, Esteve, Barcelona, Spain; 0.3 mg/sow, intramuscular) was employed as analgesic.

For embryo recovery, the reproductive tract was exposed via mid-line incision, corpora lutea were counted on the ovaries, and the embryos were collected as previously reported (19). Briefly, a small incision in the uterine wall 30–40 cm below the uterotubal junction was performed with a blunt Adson forcep, and then, a glass cannula was inserted through the incision. A volume of 30 mL of Tyrode's lactate-HEPES-polyvinyl alcohol medium (20) at 37°C was introduced into the uterine horn from the tip of the uterine horn using a 60-mL syringe connected to a blunt needle. The washing medium was forced through the glass cannula into a 50-mL sterile tube by manual massage of the uterus. The incision in the uterus was closed with continuous 2–0 polyglactin 910 sutures. Then, the uterine horns were placed back inside the abdominal cavity, and the mid-ventral incision was closed with continuous one polyglactin 910 sutures in three layers. Finally, the incision area was treated with chlorhexidine, and a single intramuscular injection of a long-acting amoxicillin suspension at a dose of 15 mg/kg was administered.

For tissue collection, three pregnant sows from each group were subjected to hysterectomies immediately after embryo recovery and the uterine tracts were transported to the laboratory on ice within 2 h of collection. The uterine horns were then opened longitudinally at the anti-mesometrial side, and samples from the ad-cervical (distal) and ad-ovarian (proximal) areas of a uterine horn were surgically collected from each sow. For histological evaluation, immediately after collection, tissue samples were fixed for 24 h in 10% neutral-buffered formalin, embedded in paraffin wax and sectioned at 4 μm. For transcriptomic analysis, endometrial tissue samples were immediately transferred to 500 μL of RNAlater (Ambion, Thermo Fisher Scientific Baltics UAB, Vilnius, Lithuania), incubated at 4°C overnight and then stored at −80°C until further use.

### Embryo Quality Evaluation

**Figure 1:**
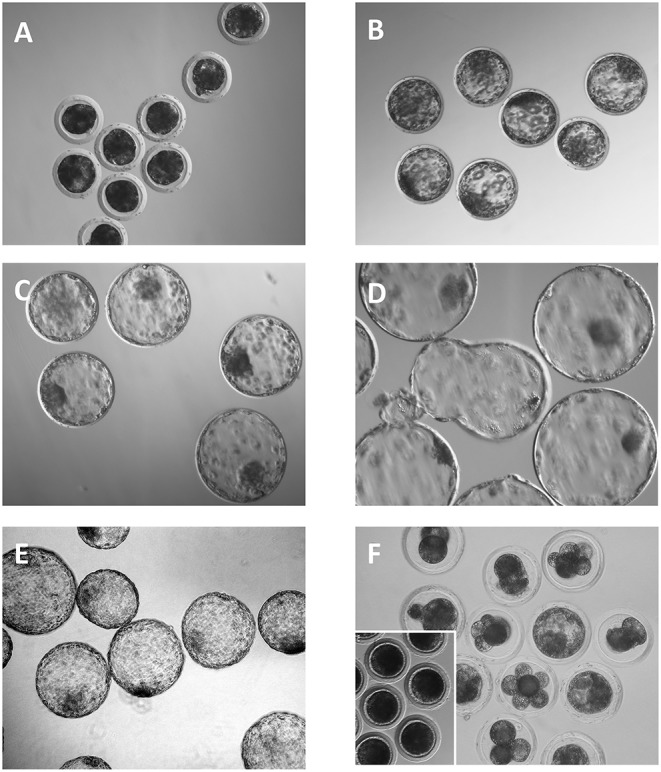
Embryos collected 6 days after the onset of estrus. **(A)** Compacted morulae. **(B)** Full blastocysts. **(C–E)** Expanded, hatching and hatched blastocysts, respectively. **(F)** Degenerated embryos. Note that most of the embryos have a developmental stage that is inappropriate for the day of the collection. Inset shows some unfertilized oocytes.

Immediately after collection, the washing medium was placed into Petri dishes and the embryos were located and assessed for morphological quality and developmental stage under a stereomicroscope. One-cell eggs and poorly developed embryos were classified as oocytes and degenerate embryos, respectively. The remaining embryos that exhibited appropriate morphology according to the criteria determined by the International Embryo Transfer Society (21) were considered viable (Figure 1). Embryos with completely compacted blastomeres, an undistinguishable cell boundary and a large perivitelline space were classified as morulae. Embryos with a differentiated blastocoel, inner cell mass and trophoblast and reduced thickness of the zona pellucida (ZP) were classified as full blastocysts. Blastocysts with increased outer diameter and very thinned ZP or with fractured or lost ZP were classified as peri-hatching blastocysts. The total cell number, as an indicator of embryo quality, was evaluated. Embryos were fixed in 4% paraformaldehyde in 0.1 M phosphate-buffered saline (PBS) for 30 min at room temperature. The embryos were then washed twice with PBS supplemented with 3 mg/mL BSA and slide-mounted in 4 μL of Vectashield (Vector Laboratories, Burlingame, CA, USA) containing 10 μg/mL Hoechst-33342 (Sigma–Aldrich Co., Madrid, Spain). Then, a small drop of vaseline was added to each corner of a coverslip, which was placed on the top of medium containing the embryos and depressed on the vaseline drops until the embryos were compressed. Embryos were analyzed by fluorescence microscopy using an excitation filter of 330–380 nm. The total cell number per blastocyst was determined by counting the number of nuclei with blue fluorescence.

### Histological Evaluation

Paraffin sections were cut and mounted on slides. The sections were deparaffinized, rehydrated, subjected to routine hematoxylin-eosin staining and examined using light microscopy (× 400) for general evaluation (22). The presence of congestion and margination of immune cells in blood vessels, edema, hemorrhage and inflammatory infiltrate in mucosal connective tissue, mitosis in cellular glands, and inflammatory cells and cellular debris in glands were evaluated and ranked as indicated in Table 1.

**Table 1:** Criteria used for the histological evaluation of the endometrium.

**Parameter**
**CongestionLeukocyte margination** +: Low number of blood vessels ++: Moderate number of blood vessels +++: High number of the blood vessels
**Edema in mucosal stromaHemorrhages in mucosal stroma** +: Focal ++: Small areas +++: Wide areas
**Inflammatory cells in mucosal stromaMitosis in cellular glandsInflammatory cells in glandsCellular debris in glands** +: Low number ++: Moderate number +++: High number

### Total RNA Extraction

Total RNA was isolated from endometrium samples using Trizol reagent (Invitrogen, Carlsbad, CA, USA) according to the manufacturer's instructions. The quantity of the obtained total RNA was measured with a NanoDrop ND-1000 (Thermo Fisher Scientific, Fremont, CA, USA). The quality of the samples was assessed using an Agilent 2100 Bioanalyzer (Agilent Technologies, Santa Clara, CA, USA). The RNA integrity number (RIN) values obtained were in the range of 8–10.

### Affymetrix GeneChip Microarray

Affymetrix Porcine Genome gene expression microarrays (Affymetrix, Santa Clara, CA, USA) were used. Microarray target sample processing, target hybridization, washing, staining and scanning steps were completed according to the manufacturer's instructions (Affymetrix, ThermoFisher Scientific, USA). Briefly, equal amounts of total RNA (250 ng) from each sample were converted to sense-strand cDNA using the one-cycle cDNA Synthesis Kit (Affymetrix) and an oligo-dT primer containing the T7 RNA polymerase promoter. *In vitro* transcription (IVT) of cRNA from cDNA was conducted following the RT-IVT method (23) using T7 RNA Polymerase. The cDNA and cRNA were purified using the Sample Cleanup Module (Affymetrix). The GeneChip IVT Labeling Kit (Affymetrix) was used for synthesis of biotin-labeled cRNA. Fragmented and labeled cDNA was loaded on the array chip (Porcine gene 1.0 ST GeneChip® Cartridge Array, Affymetrix), and hybridized during incubation at 45°C under rotation at 60 revolutions per minute, for 16 h. The hybridized array chip was then unloaded, subjected to washing and staining using a GeneChip® Fluidics Station 450 (Affymetrix), and finally scanned using the Affymetrix GeneChip® scanner GCS3000.

### Microarray Data Analysis

The array data were examined using Partek Genomics Suite 7.0 (Partek). Data were first normalized using the Robust Multichip Average RMA method (24). Differentially expressed genes (DEGs) between endometrial samples of animals treated with SP and controls were established by performing a one way-ANOVA setting parameters as a fold change (FC) >1 or < −1 with *p* < 0.05. To obtain a biologically meaningful overview of the significantly modified transcripts of the SP group relative to the control group, an enrichment analysis was performed. Analysis of overrepresented Gene Ontology (GO) terms and pathways was performed with the DAVID (database for annotation, visualization and integrated discover) and KEGG (Kyoto Encyclopedia of Genes and Genomes) databases. The Spearman's rank-correlation test was used to assess the relationship between the fold changes obtained by microarray platform and q-PCR from a total of 15 genes.

### Quantitative PCR Assay

For microarray data validation, 15 differentially expressed genes were tested by qPCR. First, total RNA from the same samples used for microarrays analysis was transcribed into cDNA using the High Capacity cDNA Reverse Transcription Kit (Applied Biosystems, Foster City, CA) with 25 mM dNTPs Mix, RT random primers, 20 U of RNase inhibitor and MultiScribe Reverse Transcriptase according to the manufacturer's protocol. All primers were designed using Primer Express™ software v3.0.1 (Applied Biosystems) and were commercially synthesized. Primer sequences are shown in Table 2. qPCR was performed with iTaq™ Universal SYBR® Green Supermix in 10-μL reactions with 50 or 150 nM for each set of primers. All reactions were carried out in a QuantStudio™ 5 Real-Time PCR System (Applied Biosystems). The thermal cycling profile was 50°C for 2 min, 95°C for 10 min, 40 cycles at 95°C for 15 s, and 60°C for 1 min. Melt curve analysis was carried out to verify the absence of primer-dimer.

**Table 2:** Primer sequences for quantitative PCR.

**Region**	**Gene symbol**	**Primers (5^**′**^-3^**′**^)**	**Accession No**.
Distal	STAT5A	F: CATCACCATTGCCTGGAAGTT R: CCTTTCACCACGCGGG	NM_214290.1
	VCAN	F: CAGGATACAGTGGCGACCAA R: CACATGCGTTGACGGTTTTAA	NM_001206429.1
	HOXB4	F: CTCAAACTATGTCGACCCCAAGT R: CGATTACCTACCCAGCGACC	ENSSSCT00000019089
	SGPL1	F: CACAGGAATGGGTGCGATCT R: CAGAATTGTCCTCCTTCTTCTTGG	XM_003133063.4
	RPL27A	F: GTCAGTGAGCAGACACGGATAAA R: CCTATCATTGACGTGGTGCG	XM_005667051.2
	TMEM200A	F: AGCCTGGATTAGGTGCAAAGA R: TGAGATTTGTTCCTCAAGCGTG	XM_001927883.6
	GBP1	F: CCAGATGACCGCCAGCTAGA R: TGAGTCCGGCACGAGTTTG	NM_001128473.1
Proximal	GNAS	F: AGCGCTCCAACGAGTACCA R: GATTGACGTCATCAAGCAGGC	NM_001130215.2
	PDGFA	F: CTGGCTGGTGGCATCCAT R: CAGAAGTCATATCAAGCTGTAGGCTG	XM_013987658.1
	DDX20	F: GGGACAGCCTCTGAGTGGAA R: AAATCCAGGTAAAGGCAAGGC	XM_001928544.4
	EIF2B2	F: TGCTGCGCCGGATCA R: TGATGGAACTGATCCGCAGA	XM_003128668.4
	TOR1A	F: TTTGACGAAATGGATAAGATGCA R: TACCTGGACTATTACGACAACCTGG	NM_001105298.1
	KCNIP2	F: TGAACTGGGCCTTCAACTTGT R: AAGGAGGAAATGCTCGACATCA	XM_001928123.3
	RPL27A	F: GTCAGTGAGCAGACACGGATAAA R: CCTATCATTGACGTGGTGCG	XM_005667051.2
	DIRAS3	F: TCTACTCCGTCACCAAGAAGCA R: ATCTGATCCGCGAGCTCAA	NM_001044598.2
Ref. gene	GAPDH	F: AGTCACAGCCCCAACTCGAT R: TTCCAGTTTCCATCCCAGACC	ENSSSCT00000000756

The qPCRs were run in triplicate per gene and per sample, and data were analyzed with the Pfaffl method to identify eventual differences in PCR efficiency. Briefly, the relative expression ratio of the target genes was calculated based on target gene efficiency (E) and the mean Ct value of the target gene in an experimental sample condition vs. the control sample one and expressed via normalization to a housekeeping reference gene (GAPDH). The corresponding gene efficiency of one cycle in the exponential phase was calculated according to the equation: E = 10^(−1/slope)^. Relative mRNA expression obtained by qPCR and microarray fold change data were compared using the Spearman's rank correlation coefficient.

### Embryonic Data Analysis

The data were analyzed with the IBM SPSS 24.0 statistics package (IBM, Chicago, IL, USA). Differences were considered significant at *P* < 0.05. The results (six replicates) are presented as the mean ± standard deviation (SD) and as percentages. Data were analyzed for normality of residuals using the Kolgomorov-Smirnov test. Data expressed as the mean ± SD (number of corpora lutea, number of embryos and oocytes collected and total cell number per embryo) were compared by an unpaired Student's *t*-test corrected for inequality of variances (Levene test). The means of the percentage data (recovery and fertilization rates) were analyzed using the Mann-Whitney test for two independent samples. Percentage data were compared using Fisher's exact test.

## Results

### Endometrium Morphology Is Affected by the Infusion of SP

**Figure 2:**
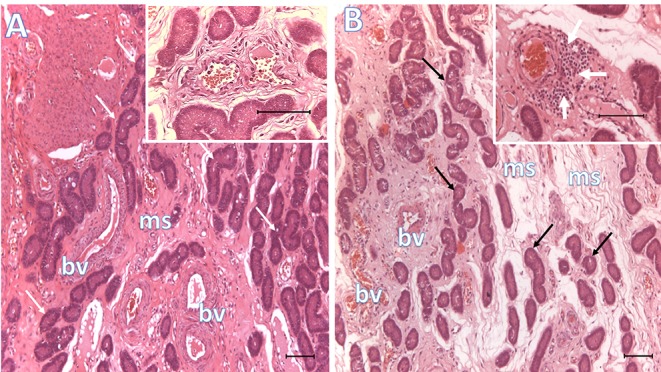
Histological representative images of the endometrium. Microphotographs of endometria 6 days after BTS (control, **A**) or heterologous seminal plasma (SP, **B**) infusions prior to AI, depicting conspicuous differences in mucosal edema, vascular congestion and peri-vascular infiltration of immune cells (see inset in **B**, white arrows; inset in **A** shows lack of peri-vascular infiltration of immune cells). Arrows, uterine glands; bv, blood vessels; ms, mucosal stroma. Bar: 100 μm.

Endometrial samples collected 6 days after heterologous SP or BTS (control) infusions prior to AI showed that inflammatory changes were accentuated in the SP group relative to endometria from the control group. These changes included congestion, leukocyte margination, edema, hemorrhages and infiltrates of immune cells in the mucosal connective tissue and the uterine glands (Table 3; Figure 2).

**Table 3:** Description of histological changes in the uterus 6 days after heterologous seminal plasma (SP) or BTS (control) infusions prior to insemination.

**Parameter**	**SP-group**	**Control group**
Congestion	+++	+/++^*^
Leukocyte margination	+++	+
Edema in mucosal stroma	++	+
Hemorrhages in mucosal stroma	+++^**^	+
Inflammatory infiltrate in mucosal stroma	+++	+
Mitosis in cellular glands	+++	+
Inflammatory cells in glands	+++	+
Cellular debris in glands	+++	+

* Depending of the zone;

** *Presence of macrophages with hemosiderin*.

### Additional Infusion of Heterologous SP Modifies the Endometrial Transcriptome Profile

**Figure 3:**
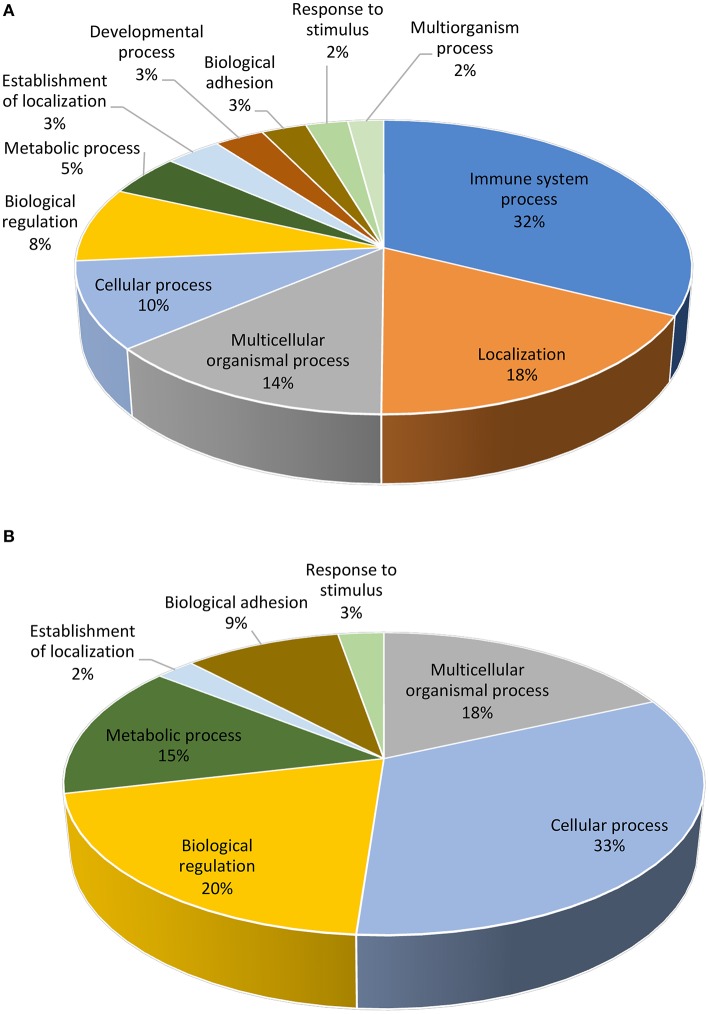
Functional distribution (KEGG database) of transcripts. The differentially expressed (*P* < 0.05) genes represented correspond to the distal **(A)** or proximal **(B)** regions of the uterine horn endometrium 6 days after heterologous seminal plasma (SP) or BTS (control) infusions prior to insemination.

Following statistical analysis of the data yield by the Affymetrix microarray, we found in the SP-group that 1,052 genes were differentially expressed in the endometrium of the distal uterine horn (514 upregulated and 537 downregulated), and 552 in the proximal uterus (245 upregulated and 307 downregulated), relative to controls. After an overall enrichment analysis, there was a conspicuous overrepresentation of biological processes associated with biological regulation, cellular processes, or immune system-related genes (Figure 3). A selection of the most significant differentially expressed biological pathways (Tables 4, 5) included pathways for cytokine-cytokine receptor interaction, MAPK-ERK signaling, TGF-beta signaling, focal adhesion, cell cycle, and cell adhesion molecules (CAMs).

**Table 4:** The most significant differently expressed biological pathways examined with KEGG database (*P* < 0.05) in the distal region of the uterine horn endometrium 6 days after heterologous seminal plasma (SP) or Beltsville Thawing Solution (BTS; control) infusions prior to insemination.

	**Pathway ID**	**Pathway name**	**Enrichment Score**	**Genes altered (%)**	Gene list^*^
1	Map04514	Cell adhesion molecules (CAMs)	1.6	4.7	**PECAM1, SLA-8, VCAN, CLDN25**, CD274, PTPRF, VTCN1
2	Map04110	Cell cycle	2.3	6.2	**ATR, ANXA1, BACH1, CRLF3, DLG1, HECA, IRF1, LIN52, ORC4, PPP1CB, SLC26A8, STAG2, STAT5A, USP37, ZPR1**, ADCYAP1, CABLES2, CHEK1, MIIP, PPP2CA, TAF2, TRIM35
3	Map04050	Cytokine-cytokine receptors interaction	0.2	2.6	**ACVR2, CRLF3, FAS, IL13**, CSF3, CXCR3, IL5, IL10RA, MAP10
4	Map04915	Estrogen signaling	0.9	3.9	**GRB2, SOS1**, ADCY4, ATF6B, GNAS, NRAS
5	Map04510	Focal adhesion	0.1	2.2	**GRB2, MRLC2, PPP1CB**, FIGF, ITGA2B
6	Map04912	GnrH signaling	0.9	4	**GRB2, SOS1**, ADCY4, GNAS, NRAS
7	Map04913	Ovarian steroidogenesis	1	4.6	**INSR**, ADCY4, GNAS
8	Map04151	PI3K-Akt signaling	0.4	3	**GRB2, INSR, LPAR1, RHEB, SOS1, PPP2R3A**, CSF3, NRAS, ATF6B
9	Map04011	MAPK-ERK signaling	2.3	8.6	**FAS, SOS1, DLG1, GRB2, LPAR1, PDCD10, RNF149**, NRAS, F2RL1, INHBE
10	Map04917	Prolactin signaling	3.7	8	**GRB2, IRF1, SOCS5, SOCS6, STAT1, STAT5A**, NRAS
11	Map04660	T-Cell receptor signaling	0.8	3.7	**DLG1, GRB2, NFKBIA**, IL5, NRAS
12	Map04350	TGF-beta signaling	0.6	3.4	**ACVR2**, INHBE, PPP2CA
13	Map04620	Toll-like receptor signaling	1.2	4.4	**LY96, NFKBIA, RIPK1, STAT1**, IRAK1, TAB1
14	Map04370	VEGF signaling pathway	0.1	1.3	NRAS
15	Map04310	Wnt signaling	0.2	2.1	**CSNK2A2, VANGL2, GRB10, KLH212, RYK, STRN**, TSC2, PTPRO, SHISA2
16	MAP04630	Jak-Stat signaling pathway	1.4	4.4	CSF3, **GRB2**, IL5, IL10RA**, IL13, SOCS5, STAT1, STAT5A**

* *Up-regulated and down-regulated genes are marked by bold and regular font, respectively*.

**Table 5:** The most significant differently expressed biological pathways examined with KEGG database (*P* < 0.05) in the proximal region of the uterine horn endometrium collected 6 days after heterologous seminal plasma (SP) or Beltsville Thawing Solution (BTS; control) infusions prior to insemination.

	**Pathway ID**	**Pathway name**	**Enrichment Score**	**Genes altered (%)**	Gene list^*^
1	Map04514	Cell adhesion molecules (CAMs)	0.2	1.2	**LRRC4C**, PVRL1
2	Map04110	Cell cycle	1.6	3.2	CCNE1, RB1, SMAD4, TP53
3	Map04050	Cytokine-cytokine receptors interaction	0.2	1.3	**PDGFA**, CCR1, CD70, LEP
4	Map04915	Estrogen signaling	2.4	3.9	**GNAS, PIK3R5**, NRAS, PIK3R2, SRC
5	Map04510	Focal adhesion	1.5	2.7	**PDGFA, PIK3R5**, ELK1, IGF1R, PIK3R2, SRC
6	Map04912	GnrH signaling	2.2	4	**GNAS**, ELK1, NRAS, SRC
7	Map04913	Ovarian steroidogenesis	1.1	3.1	**GNAS**, IGF1R
8	Map04151	PI3K-Akt signaling	2.8	3	**CDC37, FGF14, PDGFA, PIK3R5**, CCNE1, CRTC2, GYS1, IGF1R, JAK3, NRAS, PIK3R2, TP53
9	Map04917	Prolactin signaling	2.6	4.6	**PIK3R5**, NRAS, PIK3R2, SRC
10	Map04660	T-Cell receptor signaling	1.4	3	**PIK3R5**, CD247, NRAS, PIK3R2
11	Map04350	TGF-beta signaling	0.2	1.1	SMAD4
12	Map04620	Toll-like receptor signaling	0.3	1.5	**PIK3R5**, PIK3R2
13	MAP04370	VEGF signaling pathway	3	5.3	**PIK3R5**, NRAS, PIK3R2, SRC
14	Map04310	Wnt signaling	0.7	2.1	SMAD4, CSNC2B, TP53
15	Map04630	Jak-Stat signaling pathway	0.9	2.2	JAK3, LEP, PIK3R2, **PK3R5**

* *Up-regulated and down-regulated genes are marked by bold and regular font, respectively*.

**Figure 4:**
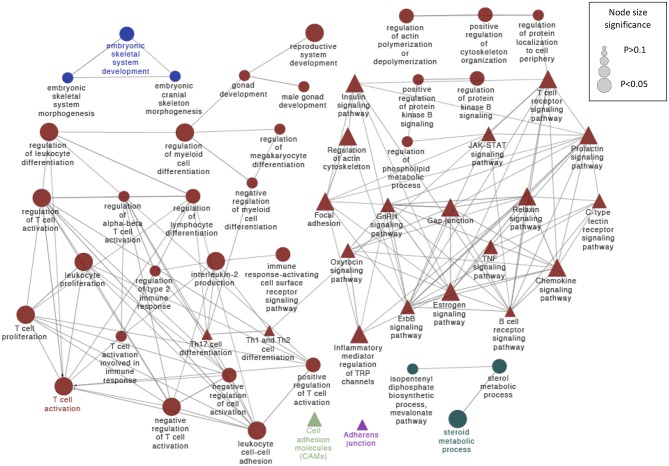
Schematic representation of selected altered transcripts potentially involved in embryo kinetics in distal and proximal regions of the SP treated endometrium relative to the BTS (control) group. The analysis of overrepresented functional categories was performed using the Cytoscape v3.0.0 application ClueGo v2.0.3. The following databases were used: GO subgroup biological process shown as circles and KEGG pathways shown as triangles. Terms are functionally grouped based on shared genes (kappa score) and are shown in different colors. The sizes of the nodes indicate the degree of significance, where the biggest nodes correspond to the highest significance. The most significant term defines the name of the group. The following ClueGo parameters were used: GO tree levels, 2–5 (first level = 0); minimum number of genes, 3; minimum percentage of genes, 5; *P*-value correction, Benjamini-Hochberg, terms with *P* < 0.05, GO term fusion; GO term connection restriction (kappa score), 0.4; GO term grouping, initial group size of 2 and 50% for group merge. The resulting network was modified; that is, some redundant and non-informative terms were deleted, and the network was manually rearranged.

From that general gene set, we selected 126 significantly altered genes involved in different biological processes and pathways, with potential roles in embryonic development, implantation, or progression of pregnancy; 84 of them were in the distal uteri and 42 in the proximal uterine regions. The most significant findings in this gene set were embryonic skeletal system development, reproductive system development, interleukin-2 production, regulation of T cell activation, and steroid metabolic process (Figure 4).

**Figure 5:**
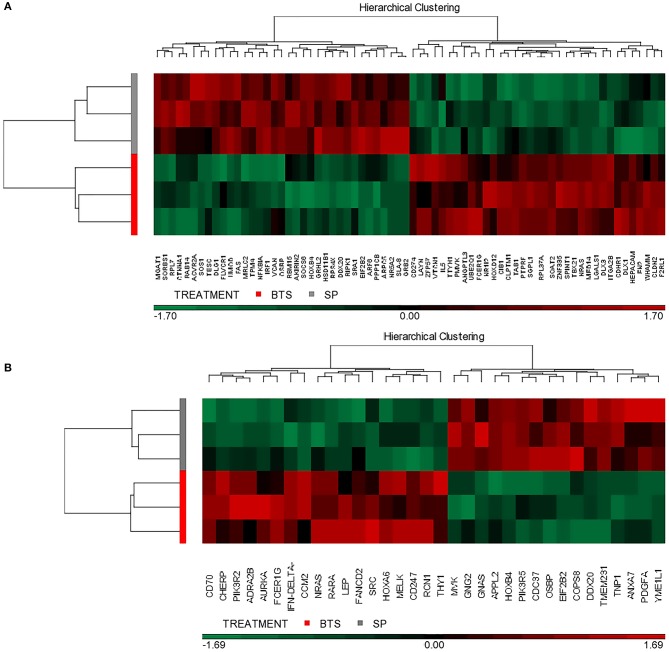
Hierarchical cluster analysis of gene expression patterns. The selected transcriptional profiles (DAVID database) were obtained from the distal **(A)** or proximal **(B)** regions of the uterine horn endometrium collected 6 days after heterologous seminal plasma (SP) or BTS (control) infusions prior to insemination. Colors correspond to relative RNA abundance for the detected genes, each of which is represented by one vertical bar. Red color indicates upregulated expression and green downregulated expression while black indicates the mean value.

Moreover, to gain insight into similarities among replicates, this particular set of genes was subjected to a hierarchical clustering procedure and presented as heatmaps (Figure 5; find gene id in this figure). The heatmap indicates that the selected differential gene set associates the biological samples correctly into two groups each representing one of the two conditions (SP vs. control).

### Validation of the Microarray Data

**Figure 6:**
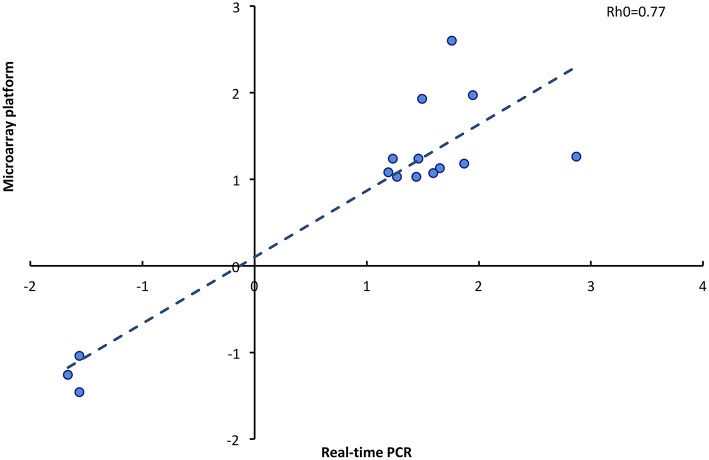
Correlation of fold change determined by microarray platform and real time PCR of 15 genes (Rho, Spearman coefficient).

To validate the microarray data, 15 genes were selected for qPCR, using the same RNA samples that were used for the microarray. The analyzed genes presented uniform patterns of expression when studied by both methods. There was a high correlation (Rh0 = 0.77; *P* < 0.001) between the fold change of genes analyzed by microarray and the fold change of genes analyzed by qPCR (Figure 6). The genes selected for real-time PCR are involved in the regulation of initial pregnancy, including embryonic development (*GNAS, SGFL1, HOXB4*), cell proliferation (*VCAN, STAT5A, PDGFA, EIF2B2*), signal transduction (*DIRAS3, DDX20*), and protein binding (*TOR1A, RPL27A, TMEM200A, KCNIP2*).

### Seminal Plasma Indirectly Affects Embryo Development

To evaluate the indirect influence of heterologous SP on embryo development, a total of 800 structures (embryos and unfertilized oocytes) recovered from 34 superovulated sows were assessed at Day 6 post-AI. The parameters evaluated are shown in Table 6.

**Table 6:** Reproductive parameters obtained 6 days after heterologous seminal plasma (SP) or Beltsville Thawing Solution (BTS; control) infusions 30 min prior to insemination.

**Group**	**No. of sows**	**Corpora lutea**	**Oocytes-embryos**	**Recovery rate (%)**	**Viable embryos**	**Fertilization rate (%)**
SP	16	25.7 ± 8.4	24.3 ± 8.6	94.8 ± 14.1	22.6 ± 7.3	94.3 ± 11.8
BTS	18	24.9 ± 5.6	22.8 ± 5.1	92.0 ± 9.7	21.1 ± 6.4	91.3 ± 14.9

*Data are mean ± standard deviation (six replicates). Means of corpora lutea, oocytes-embryos and viable embryos were compared by using unpaired Student t-test. Means of percentage data (recovery and fertilization rates) were analyzed using Mann Whitney U-test for two independent samples*.

**Figure 7:**
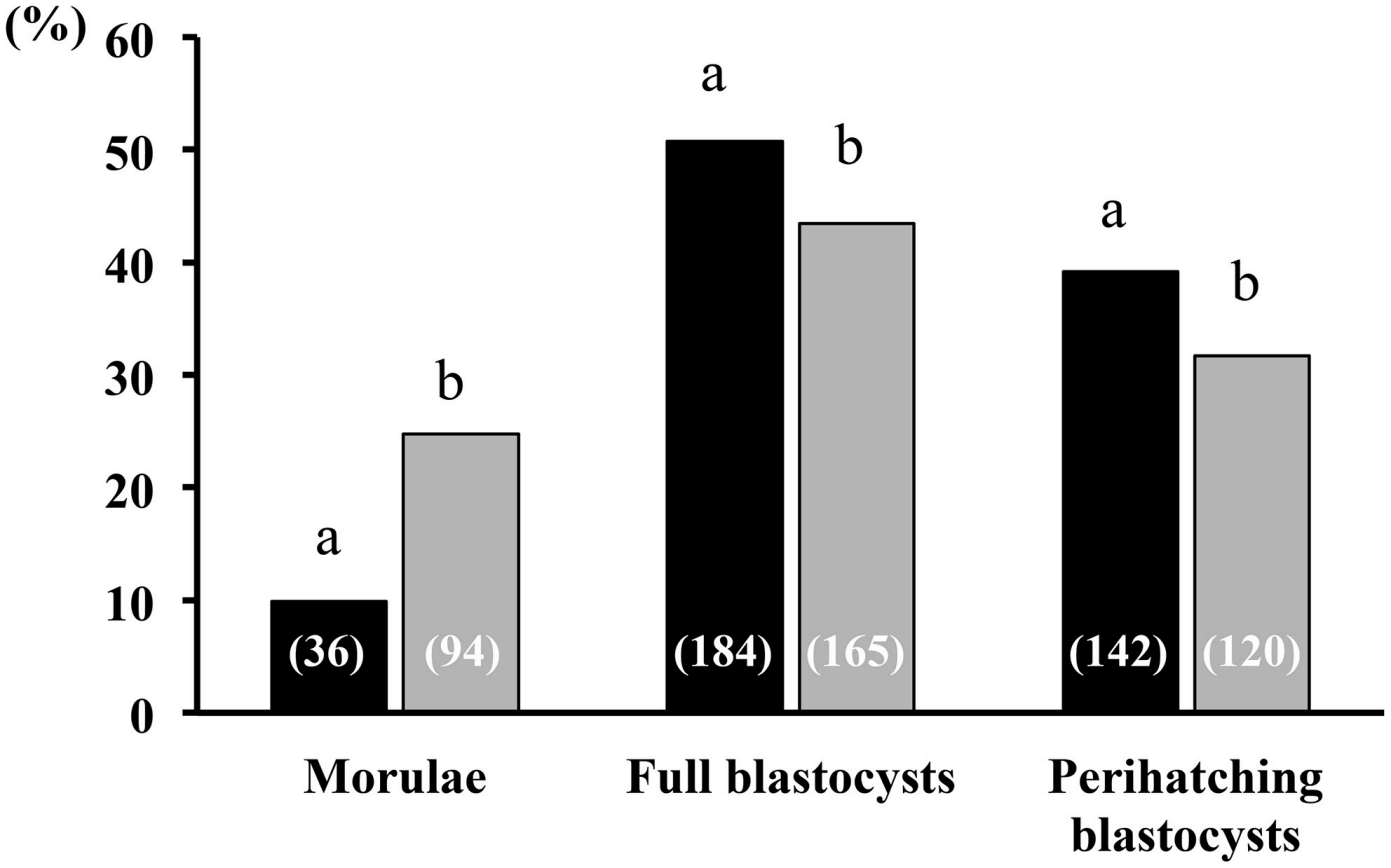
Embryonic developmental stage at collection. Developmental stages of embryo recovery 6 days after heterologous seminal plasma (SP; black bars; *N* = 16 sows) or BTS (Control; gray bars; *N* = 18 sows) infusions prior to insemination. Thirty minutes before each postcervical insemination (1.5 × 10^9^ sperm/dose in 40 mL of BTS diluent), sows were infused with 40 mL of heterologous SP or BTS (control). At day 6 after the first insemination, the sows were subjected to laparotomy to collect the embryos and to evaluate their quality and developmental stage. A total of 362 and 379 viable embryos were collected in sows from the SP and BTS groups, respectively. Numbers in parentheses are the number of embryos for each stage. ^a,b^Different letters within each embryonic developmental stage represent significant differences (*P* < 0.05; Fisher's exact test).

**Figure 8:**
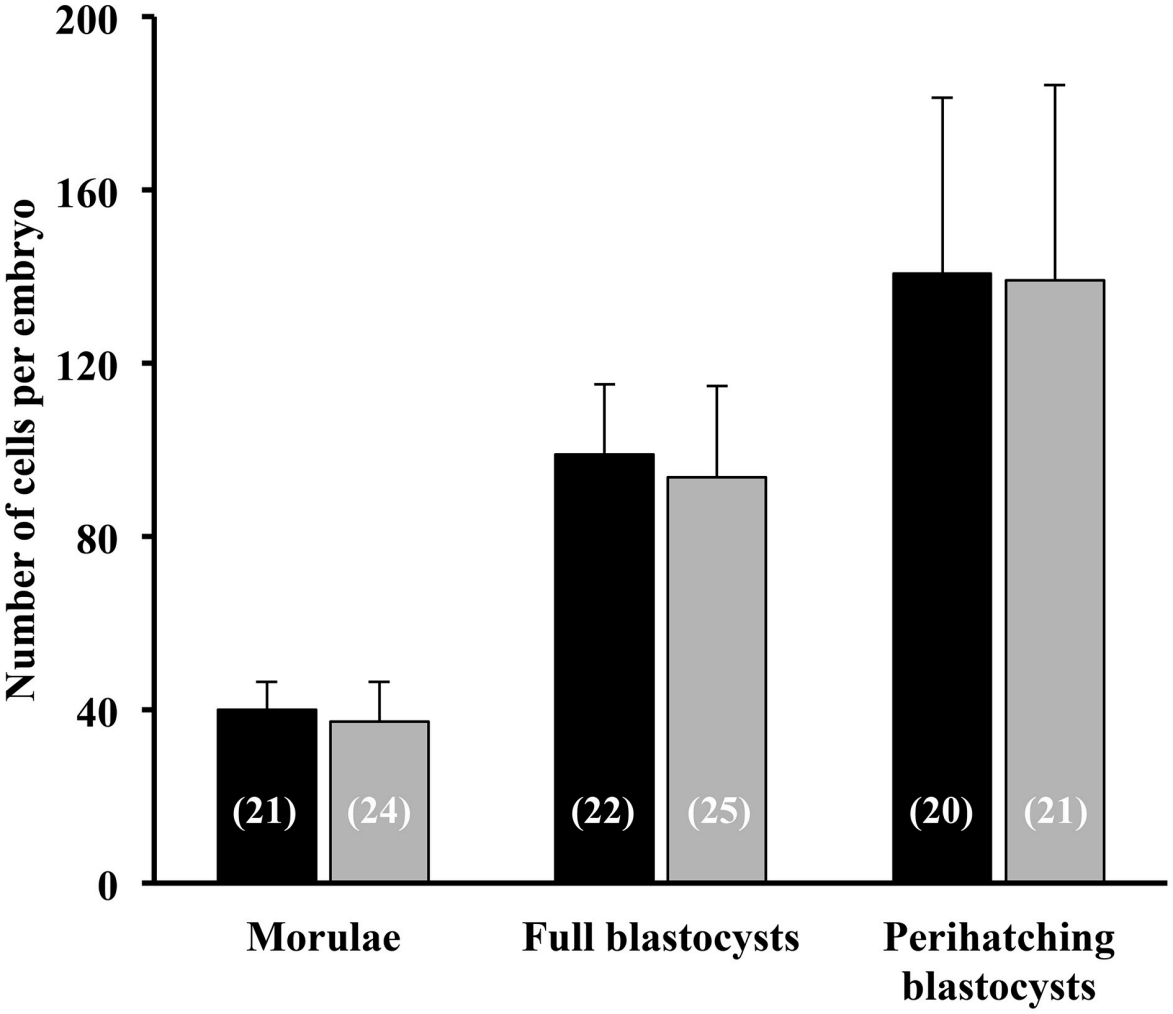
Total cell number of morulae, full blastocysts and peri-hatching blastocysts recovered 6 days after the postcervical infusion of heterologous seminal plasma (SP; black bars; *N* = 16 sows) or BTS (Control; gray bars; *N* = 18 sows) prior to insemination. Numbers in parentheses are the number of embryos counted for each stage.

All sows were embryo-bearing (“pregnant”) at the time of embryo collection. There were no differences in recovery rates between SP and control groups. The mean number of corpora lutea (CL) was also similar between groups, ranging from 16 to 49 CL and from 18 to 39 CL in the SP- and control groups, respectively. Of the recovered structures, more than 90% were classified as morphologically viable embryos, and the rest were oocytes or degenerated embryos, with no differences between groups. In total, the number of viable embryos recovered was 741, of which 17.3, 47.5, and 35.4% were morulae, full blastocysts and peri-hatching blastocysts, respectively. This embryo developmental distribution at collection varied according to the treatment used. Thus, while morulae were obtained only in 18.7% of the SP sows, in control sows the percentage was almost double (33.3%). Consequently, the sows infused with SP had fewer (*P* < 0.05) morulae and significantly (*P* < 0.05) more embryos at the full and peri-hatching blastocyst stages relative to controls (Figure 7). There were no differences in the number of total cells per embryo between the groups, within each embryonic stage (Figure 8).

## Discussion

The present study demonstrated that cervical infusion of heterologous, pooled SP prior to AI indirectly influences preimplantation embryo development in pigs. SP treatment resulted in a higher percentage of advanced stage embryos as early as 6 days post-AI relative to the control group (that solely received BTS infusions before AI).

The observed differences in embryo development could be directly related to changes in the time of ovulation in response to SP exposure. Previous studies have reported that SP modifies the endocrine-immune-cytokine network in preovulatory follicles (25, 26) leading to an acceleration of ovulation through a decrease in the interval LH peak-ovulation time-point (27). Consequently, embryos derived from heterologous SP-treated sows should be in a more advanced stage of development. However, in addition to evidence of the potential biological effects that SP induces in the ovary, it is well-known that the actions of seminal fluid can extend beyond the immediate time of exposure and become a key point for pregnancy success (28–30). Unfortunately, the number of studies exploring this subject is low. To the best of our knowledge, only one study investigated the effects of SP infusions before AI on embryo viability and developmental competence (31). These authors concluded that SP altered the dynamics of pig embryos, increasing their number and viability, and suggested that SP influenced the embryo kinetics, delaying embryo development. Our findings, in which SP did not affect embryo viability but advanced the developmental stage of the embryos strongly disagree with that report. The discrepancies may be related to the fact that those authors evaluated developmental stage by measuring embryo diameter on day 9 of pregnancy, when embryos had already hatched. It is known that a variable percentage of hatched embryos collapses or presents an irregular morphology (32), making it a non-reliable result of measurement. Moreover, as in previous studies on ET (20, 33, 34) we obtained a high embryo viability rate in the control group (>90%), which makes it difficult to detect significant differences between groups.

Multiple studies of the endometrial transcriptome during the peri-implantation period, including comparisons of transcriptomic profiles in pregnant and non-pregnant animals have been carried out. In all of these studies, alterations in the expression of genes related to the immune response have been identified (35–38). However, endometrial receptivity is also crucial for the adequate progression of pregnancy during preimplantation period and is highly influenced by processes involving changes in cytokines and other molecules secreted into the luminal fluid (39, 40). Our study focused on investigating whether differences in embryo development could be associated with changes in the uterine environment motivated by infusions of heterologous, pooled SP prior to AI. For that purpose, we performed a transcriptional analysis of the uterine endometrium collected 6 days after heterologous SP or BTS (control) infusions prior to AI. We identified the presence of ~27,000 expressed transcripts in each region (distal and proximal) of the uterine horn endometrium examined, and following statistical analysis, we identified 1,052 transcripts with differential abundance in the distal region and 552 in the proximal region when the SP treatment group was compared to the control group. We used this gene list to identify pathways and gene ontology terms modified between experimental groups.

The enrichment analysis revealed an overrepresentation of genes and pathways associated with immune processes. It is known that components in SP initiate the inflammatory cascade in the female reproductive tract during the peri-ovulatory period and induce the widely studied maternal status of immune tolerance (11), which is maintained during the course of pregnancy to allow for successful attachment to the endometrium and proper development of the fetus thereafter (41–43). Some of the most notable immune-related pathways found to be altered in our study, mostly by downregulation, were T-cell receptor signaling, TNF signaling, NK cell-mediated cytotoxicity, JAK-STAT signaling and Toll-like receptor, among others. Interestingly, a particular overrepresentation of genes associated with regulatory T cells was found. It is known that a unique subpopulation of regulatory T cells (known as T_reg_ cells) plays an essential role in attaining the status of maternal immune tolerance by acting as effective suppressors of inflammation and cell-mediated immunity (44). Therefore, these cells are fundamentally important for preventing immune rejection and thus tolerating the fetal allograft. They inhibit activation and proliferation of T cells (45, 46), prevents natural killer cell cytotoxicity (47), suppress B-cell proliferation (48), and inhibit dendritic cell and macrophage maturation and activation (49, 50) to facilitate pregnancy progression. In the absence of signals potent enough to drive T_reg_ cells at the early time of gestation, the result is faulty implantation or posterior rejection of the fetuses (51). To ensure that sufficient T_reg_ cells are present in the implantation area, their activation and proliferation during the preimplantation period are crucial. In the present study, some T_reg_-associated genes were found to be upregulated in SP-treated endometrium, including *DLG1, FAS, LGALS1, STAT5A*, and *IRF1* in the uterine horn distal region (52–56). None of these genes or others associated with T_reg_ cells were altered in the proximal uterine region. We also found an overrepresentation of the transforming growth factor-ß (TGF-ß) signaling pathway, which is especially important for inducing T_reg_ cell development, in the distal region (45). The TGF-ß superfamily plays a critical role in driving the proliferation of T_reg_ cells by modulating the function of dendritic cells (47). The present results suggest that exposure of the female genital tract to heterologous SP before AI enhance the proliferation of T_reg_ cells and impact the maternal immune response to hemiallogeneic embryos at the early gestation phase (6 days post-AI). This is of particular importance for pig ET programs to decrease the immune response of the recipient females against the fully allogeneic embryos (i.e., different parents) that are transferred.

Furthermore, several studies have reported that preimplantation embryo development and growth are profoundly impacted by uterine cytokines and growth factors secreted into the luminal fluid (57–61). At this early time of pregnancy, the embryo is free-floating in the uterus, is not in direct contact with the maternal matrix, lacks a blood supply and is extremely dependent on signals generated by these cytokines and growth factors present in the maternal environment, which either support or restrict its development as they pass through the female reproductive tract (57). Embryos express cytokine receptors from fertilization until implantation, where they play an important role in embryo metabolic function and gene expression, cell number, and viability and developmental competence (61). Some of these embryotrophic cytokines and growth factors are regulated by semen exposure (41).

Interestingly, we found many genes related to cytokine-cytokine receptor-signaling pathways that were either over- or underexpressed (repressed) in uterine samples exposed to heterologous SP. For instance, we found *CD27* and *CD70* to be downregulated in distal and proximal uterine segments, respectively. These cytokines are costimulator of TNF (tumor necrosis factor), which is an embryotoxic cytokine that induces apoptosis and hinders embryonic development and implantation (8, 62). Our results demonstrate that heterologous SP infusions downregulate the expression of these genes, which may favor embryonic developmental ability.

In addition, the enrichment analysis revealed many functional categories comprised of hormone-related genes, such as relaxin and estrogen signaling, steroid metabolic process, oxytocin signaling and more. It is well-known that embryo development and its timing are regulated by many hormones that alter the physiology to support pregnancy (43). From fertilization to subsequent embryo implantation and placentation, each phase requires hormones. The present study detected the overrepresentation of steroid- and estrogen-related genes in particular, including upregulation of *STAT5A, SRA1, OSBP*, and many protein-coding genes such as *DDX20 and INSR*, which play important roles in steroid biosynthesis, or *SOS1, GRB2, or TRIP4*, which are implicated in estrogen signaling. This indicates that the uterine environment experiences modifications in hormone control due to the presence of heterologous SP.

Moreover, our study provided a number of genes involved in cellular changes in the uterus that are related to embryonic development and implantation. The PI3K/AKT and MAPK/ERK signaling pathways happened to be upregulated in both regions of the endometrium. It is believed that these signal transduction pathways are key regulators of a number of cellular functions, including cellular proliferation and migration, mitogenesis, differentiation, anti-apoptotic processes and cell survival (63). Moreover, they have been implicated in the differentiation, migration and cytoskeletal remodeling of embryo trophoblast cells during the preimplantation period (64, 65). In addition, studies in different species have agreed that deficiencies in MAPK/ERK proteins lead to early embryonic death due to the lack of signal transduction for proliferation and invasion of trophoblasts (66–68). Another interesting pathway found to be upregulated in our study was the Wnt signaling pathway. It has been speculated that the Wnt family plays an important role in regulating many biological functions, such as embryonic development, migration, tissue homeostasis and cell proliferation, among others. Previous studies have indicated that the Wnt signaling pathways are important during the early pregnancy period (69). However, its role in early embryo development is controversial.

Some other interesting genes appeared to be highly overexpressed. For instance, *HOXB*4 was upregulated in either the distal or proximal region of the endometrium in SP group. This gene is typically expressed in porcine endometrium during early embryo development due to the presence of the embryo (70), and it is crucial for embryo segmentation (71, 72). We also observed other embryo development-related genes highly overrepresented, such as *GRHL2*, a protein-coding gene that plays an important role in establishing distinct zones of primary neurulation, regulating epithelial morphogenesis by acting as a target gene-associated transcriptional activator of apical junctional complex components (73, 74). Other upregulated genes of interest found in our study include *RAB14, MGAT1*, and *ACVR2A*, which have previously been associated with early embryo development. The *RAB14* complex is fundamental for the development of the germ layers of the fetuses (75), *ACVR2A* is involved in trophoblast proliferation, adhesion, migration and invasion (76), and lack of *MGAT1* has been related to delayed embryo development (77).

Although the adhesion of embryos to endometrium occurs later in time (Day 12–16 of pregnancy), we found many altered genes specifically involved in the regulation of cell adhesion during the pig embryo preimplantation stage, including focal adhesion and cell adhesion molecule (CAM) pathways. Since implantation requires a reciprocal interaction between the embryo and the endometrium, we suggest that constituents of SP may be responsible for enhancing implantation success as early as Day 6 when the pig embryo starts to interact with the endometrial milieu making it competent for implantation. Interestingly, previous studies have reported higher pregnancy rates when embryos develop faster (78, 79), leading to increased embryo-endometrial synchrony and implantation rates (80). In this way, pregnancy rates were reduced when embryos blastulated on Day 6 compared to embryos that blastulated on day 5 (81).

Customary high-extension of semen and hence of its seminal plasma is applied for liquid pig AI and even more dramatic for frozen boar semen. Although SP is seemingly not needed for fertility, there are data available indicating that removal of SP is of no benefit (82) for sperm motility (83), sperm intactness and viability (84), capacitation and *in vitro* fertility (85) or even contraindicated since a total removal also removed cholesterol from the sperm membrane (86). It is praxis to maintain a 10–20% of SP in pig AI semen doses, although higher proportions of 20% do not improve farrowing rates (87). Differences are also present in the effect of homologous (own SP) or heterologous (unrelated boars) SP, probably for the compensating effect of boars with higher fertility or for the differential influence of the SP from selected ejaculate fractions vs. the entire ejaculate (18, 88, 89). Noteworthy, these influences seem related to the differential presence of specific proteins (82) and/or their variable quantity (90, 91). Therefore, it might be relevant to continue studying the effect of SP over fertility outcomes, including farrowing rates and litter size, for the sake of improving breeding efficiency using AI.

In conclusion, considering all of the information above, it seems that our findings are the first to demonstrate that heterologous SP infusions prior to conventional AI influence the molecular profile of the uterine endometrium during early pregnancy. Our findings further suggest that SP infusions positively impact preimplantation embryo development and modified the expression of certain genes involved in several biological processes and pathways associated with embryonic development and maternal immune system tolerance. More research is needed to determine the real potential of SP infusion or its active constituents to maximize reproductive outcomes after AI or ET, which is of great importance for the pig industry.

## Data Availability Statement

The datasets generated for this study are available on request to the corresponding author.

## Ethics Statement

The animal study was reviewed and approved by Ethical Committee for Experimentation with Animals of the University of Murcia, Spain (research code: 638/2012).

## Author Contributions

CM, EM, MG, HR-M, CC, and MÁ-R conceived and designed the study. IP, EM, MG, and CC directed the experiments. CM, JC, IP, JR, JV, GF-D, XL, FP, EM, MG, CC, and MÁ-R performed the experiments. CM, EM, MG, HR-M, CC, and MÁ-R analyzed and interpreted the data. CM wrote the manuscript. IP, EM, MG, HR-M, CC, and MÁ-R revised and discussed the manuscript. All authors read and approved the manuscript for publication.

### Conflict of Interest

The authors declare that the research was conducted in the absence of any commercial or financial relationships that could be construed as a potential conflict of interest.
